# Rectus sheath hematoma with low molecular weight heparin administration: a case series

**DOI:** 10.1186/1756-0500-7-586

**Published:** 2014-09-01

**Authors:** Laura E J Sullivan, Dale C Wortham, Kayleigh M Litton

**Affiliations:** University of Tennessee, Knoxville 1924 Alcoa Highway, Box 56, Knoxville, TN 37920 USA

**Keywords:** Rectus sheath hematoma, Low molecular weight heparin, Anticoagulation, Enoxaparin

## Abstract

**Background:**

Rectus sheath hematoma is an uncommon but potentially serious bleeding complication that can occur spontaneously or as a result of anticoagulation administration.

**Case presentation:**

Case number one: A 62 year old chronically ill Caucasian female develops a rectus sheath hematoma seven days after hospital discharge. The previous hospitalization included low molecular weight heparin administration for deep vein thrombosis prophylaxis. The patient ultimately chooses comfort care and expires due to sepsis and respiratory failure. Case number two: A 79 year old Caucasian male develops a rectus sheath hematoma during hospital admission where LMWH is used for deep vein thrombosis prophylaxis. He is managed conservatively; however, his hematocrit drops from 46 to 25.8%. Case number three: A 44 year old chronically ill Caucasian female is treated with therapeutic low molecular weight heparin for recent deep vein thrombosis during a hospital admission. She develops a large rectus sheath hematoma requiring embolization as well as blood transfusion.

**Conclusion:**

We believe this reflects an underreported significant cause of morbidity and mortality with low molecular weight heparin administration. We review the pathophysiology of rectus sheath hematoma as well as its presentation, diagnosis, and treatment. We identify at-risk populations and proposed contributing factors. We also discuss factors leading to underreporting as well as preventive strategies implemented at our institution.

## Background

For greater than a decade, prophylactic administration of low molecular weight heparin (LMWH) has risen for hospitalized patients and is an encouraged practice of deep vein thrombosis (DVT) prophylaxis. Therapeutic dosing is considered the standard medical care for many common diagnoses such as pulmonary embolism, acute DVT, acute coronary syndrome, and atrial fibrillation [1]. Well established adverse outcomes from LMWH include gastrointestinal bleeding, intracranial hemorrhage, thrombocytopenia, and retroperitoneal bleeding [2]. Rectus sheath hematoma (RSH) is considered an uncommon bleeding complication that can occur spontaneously, after trauma, or as a result of anticoagulation therapy. Cases of RSH associated with subcutaneous LMWH injection have been reported [3–7]. We present a case series of three patients with RSH associated with subcutaneous enoxaparin injection. These were confirmed by computed tomography (CT) at our facility during a three month period. We propose that RSH may be an underreported, significant cause of morbidity and perhaps mortality in the setting of LMWH use. Diagnostic and therapeutic interventions as well as preventive strategies we have implemented at our institution are discussed.

## Case presentation

### Case number one

**Figure 1:**
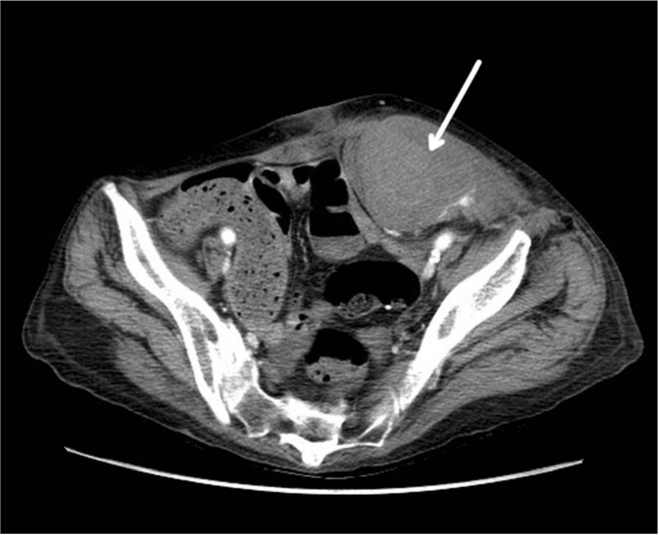
**Left rectus sheath hematoma.** A CT scan of the abdomen and pelvis revealing a large left rectus sheath hematoma (arrow).

A 62 year old Caucasian female was hospitalized for six days for treatment of a chronic obstructive pulmonary disease (COPD) exacerbation. Management included steroids and antibiotics. Home medications included clopidogrel for coronary artery disease. Her weight was 46.58 kilograms with a BMI of 17.7 and an estimated glomerular filtration rate (eGFR) of 60.65 ml/min. During the hospitalization she was treated for DVT prophylaxis with enoxaparin 40 mg by subcutaneous injection daily.Seven days after discharge, the patient returned to the emergency department complaining of diffuse abdominal pain, increasing abdominal girth, and anterior wall bruising. An abdominal CT scan confirmed a large RSH (Figure 1). Both general surgery and interventional radiology were consulted. She was managed conservatively. Her hematocrit dropped from 34.8 to 25.3%; however, she declined transfusion of blood products. During the course of her stay, the patient experienced acute hypoxic respiratory failure and did not wish to be intubated. Her medical status deteriorated and, with support from her family, the patient chose to be made comfortable with minimal supportive care. She expired due to sepsis and respiratory failure two days after re-admission.

### Case number two

**Figure 2:**
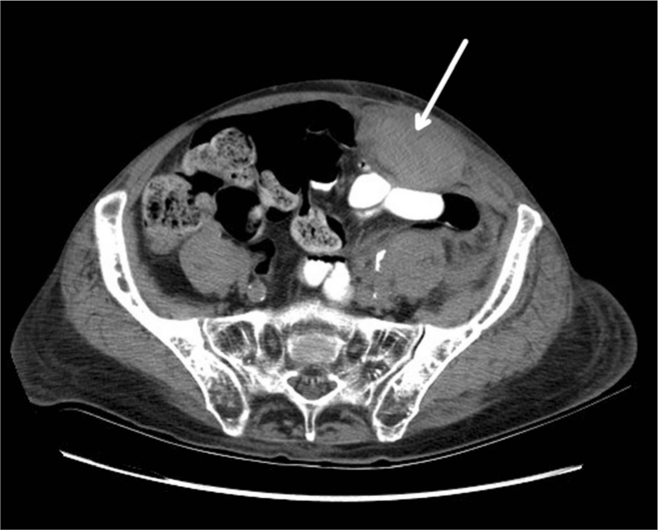
**Left rectus sheath hematoma.** A CT scan of the abdomen and pelvis revealing a large left rectus sheath hematoma (arrow).

A 79 year old male Caucasian was admitted for worsening dyspnea and management of atrial fibrillation with rapid ventricular rate and COPD exacerbation. His clinical course was further complicated by resistant gram negative and gram positive pneumonia and hypoxic respiratory failure. His weight was 59 kilograms with a BMI of 18 and initially his eGFR was 41.98 ml/min. He received subcutaneous abdominal injections of both prophylactic enoxaparin 30 mg and insulin sliding scale therapy. On day 2, his eGFR was calculated at 53.94 and his enoxaparin dose was increased to 40 mg daily.On day 5, he was noted to have abdominal pain and a noncontrast CT scan confirmed a left RSH (Figure 2) that expanded considerably over 12 hours on follow up CT. Interventional radiology was consulted but ultimately the patient was managed conservatively with fluid resuscitation and close observation. His hematocrit dropped from 46 to 25.8%. The patient was discharged after 15 days to a long term acute care facility for further management of his respiratory failure and healthcare associated pneumonia.

### Case number three

**Figure 3:**
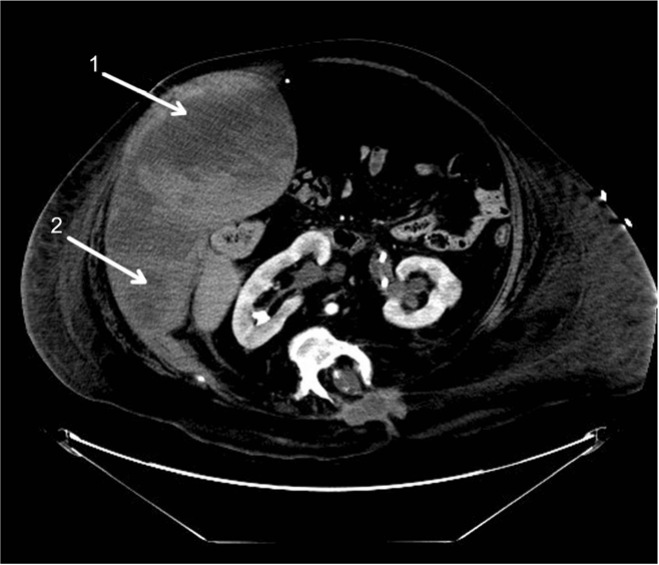
**Right rectus sheath hematoma with extension.** A CT scan of the abdomen and pelvis revealing a large right rectus sheath hematoma (arrow 1) with extension into the lateral abdominal wall (arrow 2).

An obese 44 year old Caucasian female with multiple chronic medical problems was readmitted to our facility from a ventilator capable extended care facility for sepsis and acute on chronic hypoxic respiratory failure. Comorbidities included spina bifida and central obesity with a BMI of 27. She was admitted on warfarin therapy for a history of recent deep vein thrombosis; however, she was subtherapeutic, with an International Normalized Ratio (INR) of 1.59. She was, therefore, treated with full dose enoxaparin 80 mg by subcutaneous injection twice daily. Her eGFR was 148.65 ml/min. Coumadin was held as the patient was treated with anitfungal and antimicrobial therapy that could prolong her INR.On day 8, she complained of abdominal pain and a CT scan confirmed a large right RSH (Figure 3). Repeat CT scan one day later showed extension of the hematoma and concern for possible active arterial extravasation. Both general surgery and interventional radiology were consulted. The patient underwent a failed attempt at embolization due to access complications. Due to hemorrhagic shock she required transfusion with 10 units of RBCs and pressure support ventilation in the intensive care unit. Her hematocrit dropped from 34.9 to 20.6%. Anticoagulation was reversed with fresh frozen plasma, vitamin K, and one dose of recombinant factor VIIa. The patient subsequently underwent successful embolization with thrombin and coiling of the right inferior epigastric artery by vascular surgery two days after the initial hematoma. Serial CT scans confirmed no further bleeding. After two weeks, anticoagulation therapy was reinstituted without further evidence of bleeding. The patient was ultimately discharged to a long term acute care facility for continued management of her chronic respiratory failure.

## Discussion

Rectus sheath hematoma occurs due to bleeding in the rectus sheath. This bleeding occurs either from a tear of the rectus muscle or from damage to its blood supply, including the superior and inferior epigastric arteries and branches. The rectus abdominis muscle and the epigastric arteries are surrounded by an aponeurotic sheath above the arcuate line. Below the arcuate line the posterior sheath compromises only the transversalis fascia and thus, bleeding from the inferior epigastric artery in this area can be more severe. The superior and inferior epigastric arteries anastomose around the umbilicus creating a higher concentration of vessels in this area [8, 9]. There is some evidence to suggest that aging and deconditioning of the abdominal muscles results in protuberant tissue with increased vascularity which may predispose the elderly to vessel injury and bleeding [4, 5].

Causes of RSH can include trauma, surgery, vigorous muscle contractions from coughing or straining, intraabdominal injections, and pregnancy. RSH can also occur spontaneously. Risk factors include anticoagulation therapy, cough, older age, thin body habitus, central obesity, pregnancy, female gender, recent abdominal surgery, external trauma, medical conditions causing coagulopathy, persons receiving multiple types of abdominal subcutaneous injections, and renal insufficiency [3–5, 7, 8].

The incidence of RSH is suspected to be on the rise; however, this is difficult to determine. In 1999, Klingler *et al.* found an incidence of 1.8% among 1257 hospitalized patients being evaluated by ultrasound for abdominal pain [10]. Cherry *et al.*’s case review at the Mayo Clinic found 126 patients over a 10 year period and illustrates the difficulty in determining an actual incidence rate [3]. Enoxaparin drug information provided by Lexi-Comp, Inc. reports that major hemorrhage occurs less than 1 to 4% and includes intracranial, retroperitoneal or intraocular hemorrhage. It also reports injection site hematoma as a local adverse reaction occurring at a rate of 9%. It is unclear whether RSH is grouped into the major hemorrhage or local injection site category. Lack of a ICD-9 code and lumping the diagnosis into “major bleeding” categories in large trials such as TIMI and GUSTO makes identifying the true incidence difficult [5].

Symptoms of RSH include abdominal pain, nausea, and vomiting. The symptoms can mimic conditions such as diverticulitis, appendicitis, cholecystitis, incarcerated inguinal hernia, ovarian cyst torsion, or acute pancreatitis. Physical exam often reveals a painful, firm abdominal mass corresponding to the rectus sheath. Ecchymosis does not tend to appear until 2–5 days following the hematoma [3, 4, 7, 11]. When unrecognized, RSH has resulted in inappropriate invasive procedures such as open laparotomy; however, criteria have been proposed to prevent this. Maharaj et al. recommended using physical exam maneuvers to differentiate intraabdominal from abdominal wall pathology. One such maneuver, described by Carnett in 1962, involves palpation of the tender abdomen in the supine and half-sitting positions, respectively. With this maneuver, intraabdominal processes will be most tender supine and will be protected by the contracted rectus muscle when the patient is sitting. Abdominal wall processes will remain tender in both positions [12].

Two main imaging modalities for diagnosis of RSH are ultrasound and CT. Ultrasound is not as sensitive as CT; however, it is typically more rapid and does not expose the patient to radiation. CT is useful because it is 100% sensitive and specific and can determine the presence of active bleeding [10, 13]. In order to better classify radiologic findings and suggest appropriate management strategies for findings documented, Berna et al. has described a classification system of RSH based on CT results. This classification system is divided into three types of RSH. Type I TSH represents an intramuscular, unilateral hematoma not dissecting into a fascial plane. Type II RSH represents an intramuscular, unilateral or bilateral hematoma which does not dissect into the fascial plane, but without occupation of the prevesical space. Type III RSH may or may not involve muscle, and blood can be seen within the transversalis fascia, peritoneum, and prevesical space [14].

Treatment of Type I and Type II RSH is usually conservative. Treatment of Type III RSH frequently involves blood transfusion [14]. Invasive treatment includes angiography with embolization or surgical management with exploration, hematoma evacuation, and ligation of bleeding vessels; however, this is only indicated if the RSH is progressive or if the patient is hemodynamically unstable despite adequate resuscitation [13]. Morbidity related to RSH includes all required interventions mentioned here as well as prolonged hospital stay and increased healthcare costs. Mortality data is difficult to determine given the lack of data tracking [3, 7, 15].

The association between RSH and LMWH is not well understood. The SYNERGY trial has demonstrated an increased bleeding risk when switching from one type of heparin product to another (i.e. from unfractionated heparin to LMWH), but this was not a contributing factor in our cases [15]. Injection technique has been implicated in numerous case reports [3–7]. The Lovenox® (Sanofi-aventis U.S., Bridgewater, NJ) package insert advises that the injection sites should alternate between the left and right anterolateral and left and right postero-lateral abdominal wall, avoiding the umbilicus by at least two inches. There is a warning not to inject intramuscularly. Instructions also state that the whole length of the needle should be introduced into a skin fold and advise that the skin fold should be held throughout the injection interval. Consideration of alternate injection sites such as the posterior deltoid has been suggested for higher risk patients [5, 12].

The facts of these cases were reviewed for quality improvement and were presented at a morbidity and mortality conference at our facility. This led to a systems review and re-education of nursing staff, including injection technique, site selection, and identification of higher risk patients. In addition, the eGFR is now calculated and reported with basic lab work, allowing for ease of renal dose adjustments.

## Conclusion

A series of cases at our institution highlight RSH as a serious potential complication of subcutaneous LMWH administration. Complicated coding schematics, with no existing ICD-9 code for the purpose of documentation and tracking of such events, may attribute to underreporting of RSH in both the inpatient and outpatient setting. Diagnosis of RSH can be accomplished with ultrasound or CT and management of RSH is usually conservative.

We encourage other practitioners and institutions to be aware of RSH and support the emphasis on staff and caregiver training as an important factor in its prevention. Recognition of higher risk patients, careful injection site selection, and proper injection technique should be included in this training.

## Consent

For case number one, written, informed consent was obtained from the patient’s next of kin for publication of this case report and any accompanying images. A copy of the written consent is available for review by the Editor-In-Chief of this journal.

For case numbers two and three, written, informed consent was obtained from the patients for publication of these case reports and accompanying images. A copy of the written consent is available for review by the Editor-in-Chief of this journal.

## Abbreviations

LMWH: Low molecular weight heparin
DVT: Deep vein thrombosis
RSH: Rectus sheath hematoma
CT: Computed tomography
COPD: Chronic obstructive pulmonary disease
eGFR: Estimated glomerular filtration rate
INR: International normalized ratio.
